# Identification of differentially expressed genes in flower, leaf and bulb scale of *Lilium* oriental hybrid ‘Sorbonne’ and putative control network for scent genes

**DOI:** 10.1186/s12864-017-4303-4

**Published:** 2017-11-22

**Authors:** Fang Du, Junmiao Fan, Ting Wang, Yun Wu, Donald Grierson, Zhongshan Gao, Yiping Xia

**Affiliations:** 1 College of Horticulture, Shanxi Agricultural University, Taigu, 030801 China; 2 Department of Horticulture, College of Agriculture & Biotechnology, Zhejiang University, Hangzhou, 310058 China; 3 Plant & Crop Sciences Division, School of Biosciences, University of Nottingham, Sutton Bonington Campus, Loughborough, LE12 5RD UK

**Keywords:** Gene expression, Organogenesis, *Lilium*, Transcriptome, RNA-seq

## Abstract

**Background:**

Lily is an economically important plant, with leaves and bulbs consisting of overlapping scales, large ornamental flowers and a very large genome. Although it is recognized that flowers and bulb scales are modified leaves, very little is known about the genetic control and biochemical differentiation underlying lily organogenesis and development. Here we examined the differentially expressed genes in flower, leaf and scale of lily, using RNA-sequencing, and identified organ-specific genes, including transcription factors, genes involved in photosynthesis in leaves, carbohydrate metabolism in bulb scales and scent and color production in flowers.

**Results:**

Over 11Gb data were obtained and 2685, 2296, and 1709 differentially expressed genes were identified in the three organs, with 581, 662 and 977 unique DEGs in F-vs-S, L-vs-S and L-vs-F comparisons. By functional enrichment analysis, genes likely to be involved in biosynthetic pathways leading to floral scent production, such as *1-deoxy-D-xylulose-5-phosphate synthase* (*DXS*), *3-ketoacyl-CoA thiolase* (*KAT*), *hydroperoxide lyase* (*HPL*), *geranylgeranyl pyrophosphate* (*GGPP*) *4-hydroxy-3-methylbut-2-en-1-yl diphosphate* (*HDS*) and *terpene synthase* (*TPS*), and floral color genes, such as *dihydroflavonol 4-reductase* (*DFR*), *chalcone synthase* (*CHS*), *chalcone isomerase* (*CHI*), *flavonol synthase* (*FLS*) were identified. Distinct groups of genes that participate in starch and sucrose metabolism, such as *sucrose synthase* (*SS*), *invertase* (*INV*), *sucrose phosphate synthase* (*SPS*), *starch synthase* (*SSS*), *starch branching enzyme* (*SBE*), *ADP-glucose pyrophosphorylase* (*AGP*) and*β-amylase* (*BAM*) and photosynthesis genes (*Psa*, *Psb, Pet and ATP*) were also identified. The expression of six floral fragrance-related DGEs showed agreement between qRT-PCR results and RPKM values, confirming the value of the data obtained by RNA-seq. We obtained the open reading frame of the *terpene synthase* gene from *Lilium* ‘Sorbonne’, designated *LsTPS*, which had 99.55% homology to transcript CL4520.Contig5_All. In addition, 54, 48 and 50 differently expressed transcription factor were identified by pairwise comparisons between the three organs and a regulatory network for monoterpene biosynthesis was constructed.

**Conclusions:**

Analysis of differentially expressed genes in flower, leaf and bulb scale of lily, using second generation sequencing technology, yielded detailed information on lily metabolic differentiation in three organs. Analysis of the expression of flower scent biosynthesis genes has provided a model for the regulation of the pathway and identified a candidate gene encoding an enzyme catalyzing the final step in scent production. These digital gene expression profiles provide a valuable and informative database for the further identification and analysis of structural genes and transcription factors in different lily organs and elucidation of their function.

**Electronic supplementary material:**

The online version of this article (10.1186/s12864-017-4303-4) contains supplementary material, which is available to authorized users.

## Background

The processes whereby an organism develops its shape and the cells and organs take on specific metabolic roles and structural characteristics are essential aspect of development and differentiation. The analysis of mutants has been a very productive approach to understand these processes and identify important regulatory elements since the last Century and several key genes that control plant organogenesis have been identified from different crops using this approach. For example, the ABC floral organ identity genes [1], *LeHB-1*, that participates in regulating tomato floral organogenesis [2], *lic-1*contributing to plant architecture [3], and *ZmGSL* which plays a role in early lateral root development [4]. However, the analysis of mutants is time- and labor-consuming, and particularly difficult in plants with large genomes. With the development of biotechnology, the establishment of full genome-sequences has dramatically increased our knowledge of plant genetics and molecular biology [5]. Sequencing the expressed part of the genome is nowadays achievable, even for plants such as lily with large genomes (~36Gb) [6, 7], and this can reduce the complexity and provide useful information and tools for molecular analysis [7, 8], enabling the identification of important genes.

Lilies are highly prized monocotyledonous plants (family Liliaceae, genus *Lilium*) with prominent bulbs, linear or oval leaves with parallel veins, and ornamental flowers. Although the taxonomy of lilies is complex, the Illumina sequencing-based digital gene expression (DGE) profiling technology, also called RNA-seq technology, has recently been applied extensively in lily research. In the past three years a large volume of data about differentially expressed genes (DEGs) involved in vernalization [9], cold-stress response [10], carbohydrate metabolism [11], dwarfism [12], pollen germination [13], flower development [14], flower color biosynthesis [15, 16] have been generated and analyzed in lily. In 2011, a few genes for developmental traits of lily were located on the genetic map, for example for flower colour (*LFCc*), flower spots (*lfs*), stem colour (*LSC*), antherless phenotype (*lal*) and flower direction (*lfd*) [17].

Lily bulbs consist of imbricating scales, which are modified swollen leaf bases and the large flowers have six petaloid tepals, six stamens and a superior ovary. Flowers are characterized by showy color and fragrance which are both economically important and essential for attracting pollinators in the natural environment and bulb scales are rich in starch and are important storage organs in the dormant bulb. All organs of a lily originate from the basal plate of a bulb, which makes lily morphogenesis particularly interesting. However, there have been no reports of studies on gene expression during lily organogenesis. To develop SSR markers, we analyzed a hybrid assembly transcriptome database (L.-Unigene-All) from lily (accession number: SUB2623518), based on the Illumina HiSeq 2000 sequencing platform. Taking this as a reference sequence, we carried out a digital gene expression profiling of flowers, leaves and bulb scales of *Lilium* oriental ‘Sorbonne’, and identified DEGs, including transcription factors and structural genes, in the three organs for the first time, providing insights into the genes participated in the differentiation of these organs, focusing mainly on candidate genes putatively participating in flower color, scent production, leaf photosynthesis and bulb development. Quantitative real-time PCR (qRT-PCR) and gene cloning were carried out for selected candidate genes involved in scent production to verify the conclusions from RNAseq data and identify genes for future analysis.

## Results

### Summary of gene expression profiling

Three replicates for each organ were used for cDNA library construction. For one leaf sample 74.78% of the reads matched Tulip virus X (Additional file 1: Table S1) and the data for this sample were discarded. Overall, we obtained over 11 Gb data for lily flower, leaf and scale organs. From these reads, between 73.49 and 83.12% from each sample could be mapped to the reference transcriptome. There were approximately 6 Mb perfect reads for each organ, accounting for more than 67.05% of those that were mapped to the reference transcriptome (Table 1), excluding reads with more than 2 bp mismatches. Approximately 77.71% of these sequences matched to the reference transcriptome were unique matched reads, with some multi-position reads. These data, which lay a valuable cornerstone for future work, have been submitted to the NCBI SRA database (accession number: SRP084220).

**Table 1:** Summary of gene expression profiling for three lily organs

Organ	Total reads	Total mapped Reads	Perf. match	≤2 mismatch	Unique match	Multiposition match
Flower	11,877,133	9,781,891 (82.36%)	6,639,595 (67.88%)	3,142,296 (32.12%)	8,163,994 (83.46%)	1,617,897 (16.54%)
	11,449,580	9,216,439 (80.50%)	6,350,723 (68.91%)	2,865,716 (31.09%)	7,136,152 (77.43%)	2,080,287 (22.57%)
	11,349,225	8,959,443 (78.94%)	6,040,856 (67.42%)	2,918,587 (32.58%)	7,444,467 (83.09%)	1,514,976 (16.91%)
Leaf	11,481,948	8,894,809 (77.47%)	5,680,563 (63.86%)	3,214,246 (36.14%)	6,766,819 (76.08%)	2,127,990 (23.92%)
	11,708,654	9,732,816 (83.12%)	6,914,703 (71.05%)	2,818,113 (28.95%)	6,977,857 (71.69%)	2,754,959 (28.31%)
Scales	11,682,968	8,902,142 (76.20%)	5,830,385 (65.49%)	3,071,757 (34.51%)	6,859,013 (77.05%)	2,043,129 (22.95%)
	11,101,394	8,158,589 (73.49%)	5,571,981 (68.30%)	2,586,608 (31.70%)	6,333,245 (77.63%)	1,825,344 (22.37%)
	10,552,892	8,566,038 (81.17%)	5,440,069 (63.51%)	3,125,969 (36.49%)	6,448,796 (75.28%)	2,117,242 (24.72%)

### Analysis of DEGs in each organ

Both RPKM value and false discovery rate were used for DEGs screening and original values of all transcripts are shown in Additional file 2: Table S2. Of the millions of gene sequences expressed in each biological sample, some transcripts were present in all three organs, with different expression levels but only a few thousand were significantly differentially expressed genes (DEGs) unique to a specific organ with undetectable expression in the other organs.

**Fig. 1:**
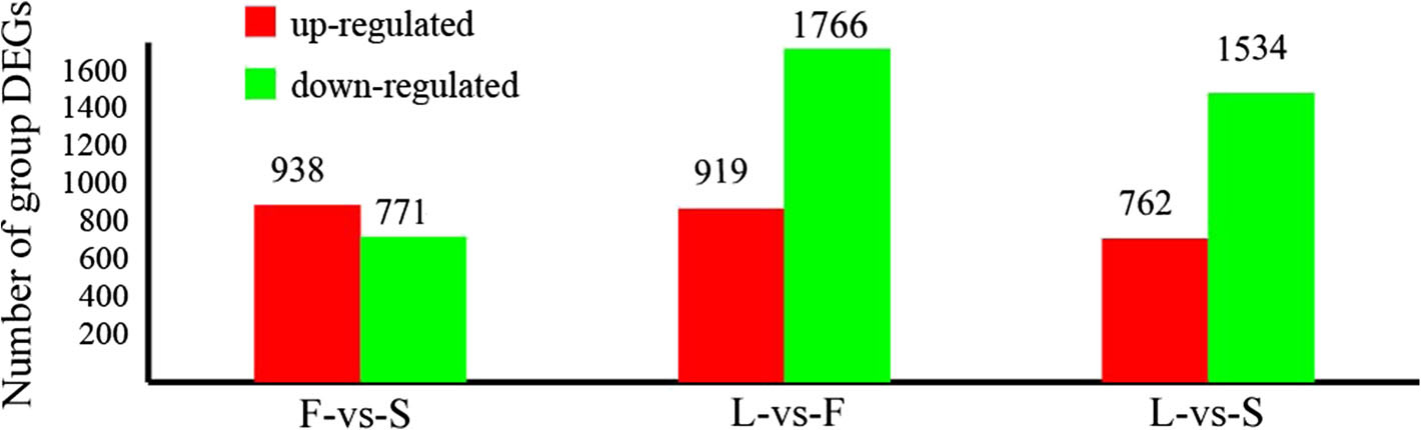
Number of differentially expressed genes in leaf (L), flower (F) and bulb scale (S)

We obtained 2685 DEGs in the comparison of leaf-vs-flower (L-vs-F), 2296 in leaf-vs-scale (L-vs-S), and 1709 in flower-vs-scale (F-vs-S), with a false discovery rate value <0.001 [18]. From the F-vs-S comparison, there were more up-regulated genes (938) than down-regulated genes (771) in scales, taking flower as a reference organ, while with leaf as a reference organ, more genes were down-regulated in flower (1766) and scale (1534) (Fig. 1), indicating a greater number of transcripts of DEGs (65.77–66.81%) differentially expressed in leaf compared with flower and scale. There were 73 DEGs expressed in three comparisons (Additional file 3: Table S3). Thirty-six percent of these genes common had putative functions in photosynthesis and the others participated in basic processes, such as metabolism of carbohydrates, nitrogen and proteins. Two genes, CL1446.Contig13_All and Unigene6258_All, related to pathogene and defensin, were found in the three organs (Additional file 3: Table S3).

Taking uniquely expressed genes as those with an expression value of zero in two organs while having expression value more than zero in the third organ, 474, 825 and 404 unique genes were found for flower, leaf and scale, respectively. Of these, approximately 70% have been annotated (Additional file 4: Table S4, Additional file 5: Table S5 and Additional file 6: Table S6) and there are twice the number of unique genes in leaf compared to flower and scale (Table 2).

**Table 2:** Number and distribution of annotated unique organ genes

Organ	Unique gene	Annotated	Unannotated	Annotation percent (%)
Flower	474	324	150	68.4
Leaf	825	439	386	53.2
Scale	404	286	118	70.8

### Gene ontology analysis of significantly DEGs

**Fig. 2:**
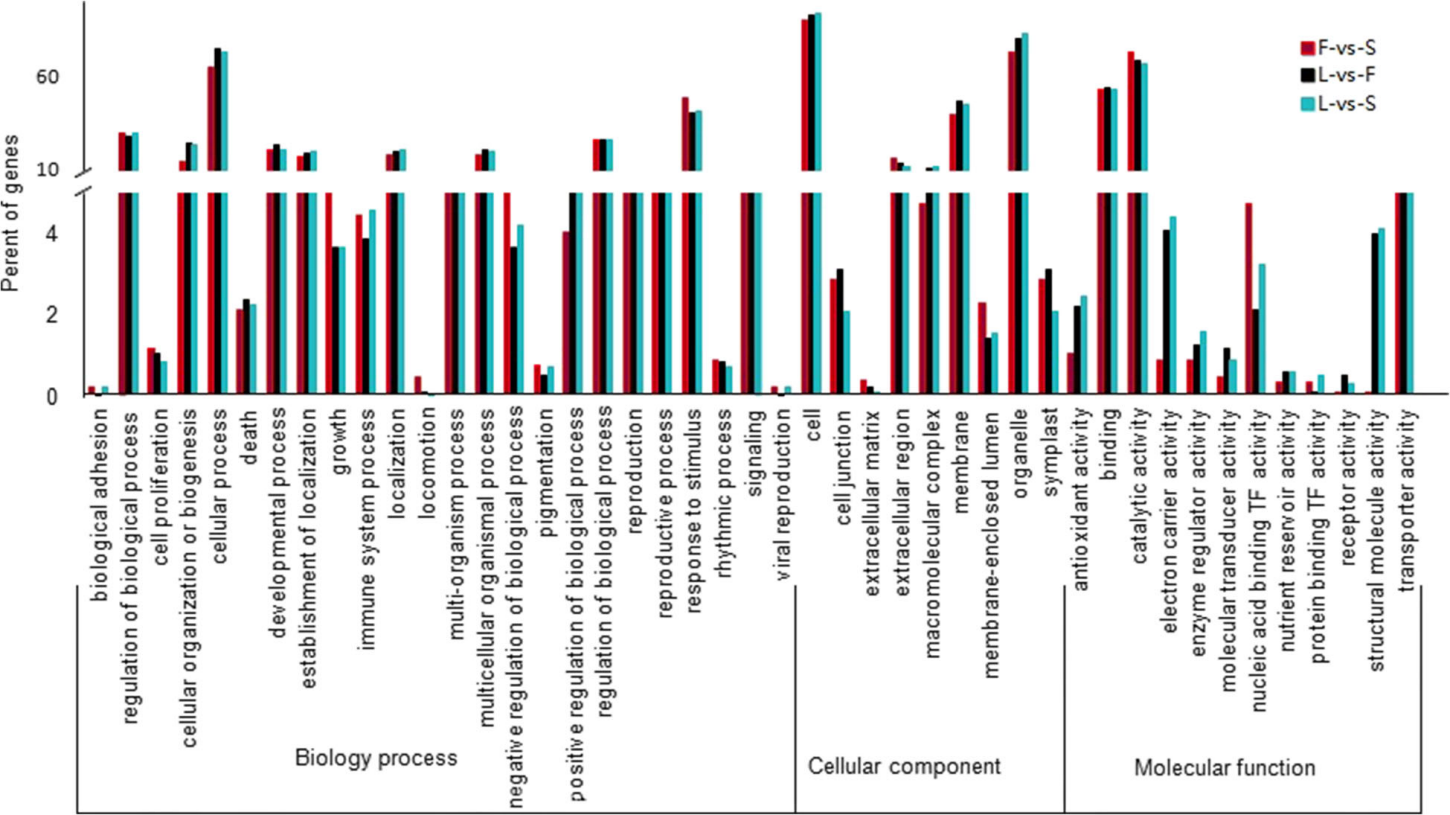
Functional categorization of genes differentially expressed in three pairwise comparisons of leaf (L), flower (F) and scale (S)

Approximately 17% of differentially expressed genes were unannotated in the database (296 in F-vs-S, 466 in L-vs-F and 393 in L-vs-S), suggesting that at least some might be new unknown transcripts, and further work will be necessary to investigate their roles. The majority (83%) of the DEGs had annotations indicating a likely function, and the numbers and functions of genes in the three pairwise comparisons were similar (Fig. 2), with 24 functional groups classified under biology process, 9 under cellular components and 12 functional groups in molecular functions. In the biological process category, most genes were characterized as having functions in categories “cellular process” (GO: 0009987), “response to stimulus” (GO: 0050896) or “regulation of biological process” (GO: 0050789), and in the cellular component subgroup, most genes were classified as related to “cell” (GO: 0005623), “organelle” (GO: 0043226) and “membrane” (GO: 0016020). Most genes in the molecular function category encoded proteins with “catalytic activity” (GO: 0003824), “binding” (GO: 0005488) and “transporter activity” (GO: 0005215) (Fig. 2).

### KEGG pathway analysis of significantly DEGs

**Fig. 3:**
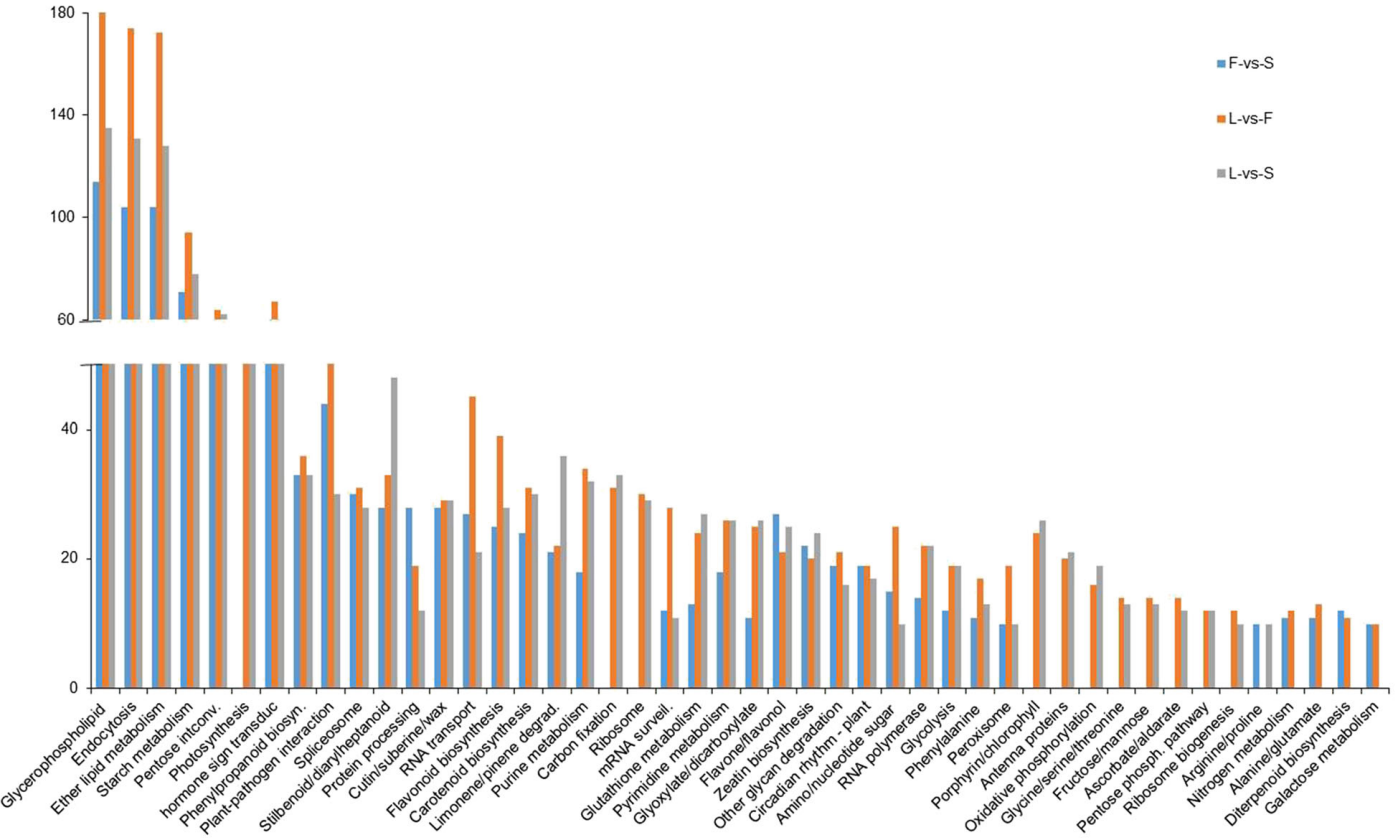
Comparison of KEGG pathways with more than 10 DEGs in three pairwise comparisons of leaf (L), flower (F) and bulb scale (S)

KEGG (Kyoto Encyclopedia of Genes and Chromosomes) pathway enrichment analysis was used to identify DEGs significantly associated with specific metabolic pathways. A total of 1461 DEGs from the L-vs-F comparison were assigned to 117 KEGG pathways; for F-vs-S comparison, 885 DEGs had 109 pathway annotations; and in the L-vs-S comparison 1194 DEGs were related to 105 pathways. There were more DEGs with pathway annotations for L-vs-F than for the F-vs-S and L-vs-S comparisons. Genes for 95 pathways were expressed in all three organs and included housekeeping activities. More than 40% of the DEGs participated in metabolic pathways, and around 20% in the three comparisons were related to biosynthesis of secondary metabolites. In addition, more than 10% of DEGs in three pairwise organ comparisons were involved in pathways of glycerophospholipid metabolism, endocytosis and ether lipid metabolism. Figure 3 lists 46 KEGG pathways where there were more than 10 DGEs between the three pairwise comparisons. There were no DEGs in 11 KEGG pathways in the F-vs-S comparison, and these were mainly pathways involved in photosynthesis and carbohydrate and protein metabolism. For L-vs-S comparison four KEGG pathways showed no DEGs, including nitrogen metabolism, alanine/glutamate metabolism, diterpenoid biosynthesis and galactose metabolism.

### Mining of candidate genes related to flower color, fragrance, photosynthesis and bulb development

By GO and KEGG annotation, 20 up-regulated flower color genes were found with putative functions in flavonoid biosynthesis, including sequences homologous to *DFR (dihydroflavonol 4-reductase), CHS (chalcone synthase), CHI (chalcone isomerase), FLS (Flavonol synthase), F3H (flavanone 3-hidroxylase), LAR (leucoanthocyanidin reductase), HCT (shikimate O-hydroxycinnamoyltransferase), LDOX (leucoanthocyanidin dioxygenase), TT7 (flavonoid 3′-monooxygenase)* (Additional file 7: Table S7) and some of those genes, such as *DFR* and *CHS,* showed more than 10 fold higher expression level in flower compared to leaf and scale.

Monoterpenoids are the dominant classes of volatile compounds emitted from scented lilies [19–21] and genes encoding TPSs (terpene synthases) (Additional file 8: Table S8) involved in monoterpenoids biosynthesis were identified in both L-vs-F and F-vs-S comparisons. From comparisons of the predicted amino acid sequence with those of known genes from *Freesia hybrid* and *Litsea cubeba*, these were predicted to encode linalool and ocimene synthases, respectively. These *TPSs* were significantly up-regulated in flowers as were some other genes encoding enzymes participating in the 2-C-methyl-D-erythirtol 4-phosphate (MEP) pathway, which leads to the synthesis of monoterpenes, including *DXS (1-deoxy-D-xylulose-5-phosphate), GGPP (geranylgeranyl pyrophosphate)* and *HDS (4-hydroxy-3-methylbut-2-en-1-yl diphosphate)* (Additional file 5: Table S5). We also investigated *HPL (hydroperoxide lyase)* and *KAT (3-ketoacyl-CoA thiolase)* (Additional file 8: Table S8), which participate in the oxylipin metabolism and β-oxidation pathways, as these genes were highlighted in a recent lily floral fragrance study as being differentially expressed in lily cultivars [20]. Both *HPL* and *KAT* which were up-regulated in flower compared with scale and leaf.

Sixty-three DEGs involved in photosynthesis were identified as being up-regulated, in the transcriptome of mature leaves, related to photosystem, photosystem II, the cytochrome b6/f complex, photosystem electron transport and F-type ATPase (Additional file 9: Table S9).

Genes involved in carbohydrate metabolism, especially starch and sucrose, were of particular interest in this study because of their importance for bulb function. Twenty-one DEGs, 11 in L-vs-S and 11 in F-vs-S comparisons (with one in common for both L-vs-S and F-vs-S), were predicted to participate in metabolism of starch and sucrose (Additional file 10: Table S10). Key genes homologous to *SS (sucrose synthase), INV (invertase), SPS (Sucrose phosphate synthase), SSS (starch synthase), SBE (starch branching enzyme), AGP (ADP-glucose pyrophosphorylase)* and *BAM (amylase)* were identified. Most genes, putatively related to sucrose and starch hydrolysis, were down-regulated in scale relative to flower as reference. Different genes putatively related to sucrose or starch synthesis and hydrolysis were either up-regulated or down-regulated in scales with leaf as reference (Additional file 10: Table S10), presumably related to complicated metabolic differences in bulb at this developmental stage.

### Expression patterns of genes involved in monoterpene biosynthesis

**Fig. 4:**
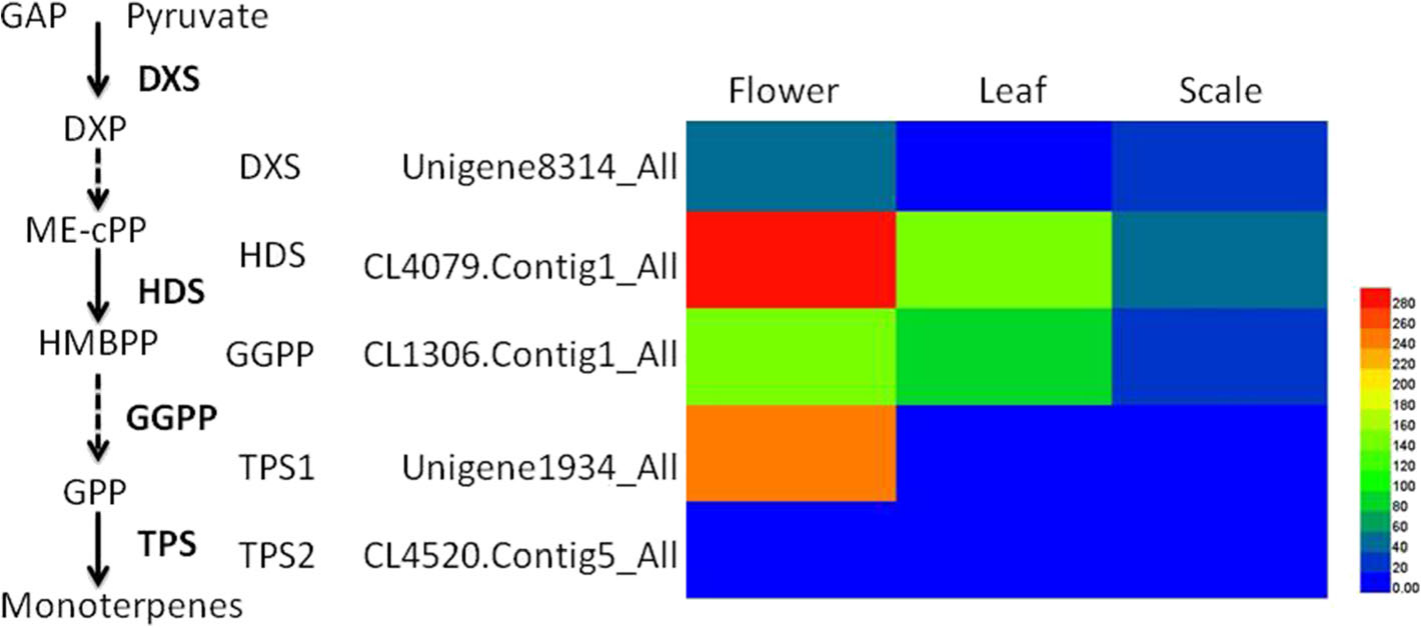
Heatmaps of DEGs related to monoterpene biosynthesis

Flower scent production is of immense importance in plant biology and ecology. It plays a role in reproduction by promoting pollination through interactions with insects and other organisms, and it also contributes value to many commercially important horticultural flowers, such as *Lilium* cv. Sorbonne. Many scents are produce from the terpenoid pathway, as are many other biology important molecules. Therefore, we investigated genes encoding enzymes involved in monoterpene biosynthesis. In this study, five DEGs encoding four putative enzymes related to monoterpene biosynthesis were identified from the transcriptome (Additional file 8: Table S8). Genes *DXS* (Unigene8314_All), *HDS* (CL4079.Contig1_All), *GGPP* (CL1306.Contig1_All) and *TPS1* (Unigene1934_All) showed significantly higher expression in flower than in leaf and scale (Fig. 4), whereas *TPS2* showed extremely low expression in three organs.

### Transcription factor analysis

**Fig. 5:**
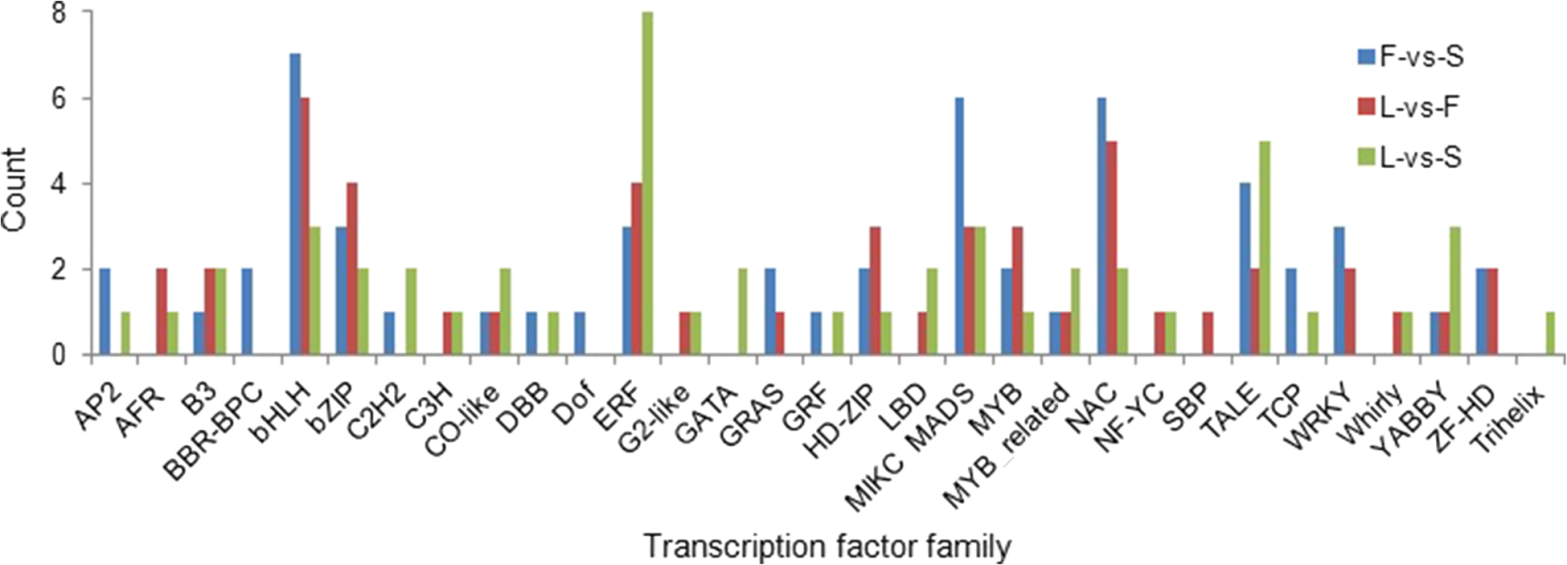
Distribution of putative transcription factors identified for three pairwise comparisons of leaf (L), flower (F) and bulb scale (S)

Transcriptome unigenes of L.-Unigene-All were searched against the Plant Transcription Factor Database and 839 unigenes identified as transcription factors (TFs) sequences belonging to 53 putative transcription factor families, with the three largest groups being bHLH (75), MYB related (60) and C3H (55). These putative transcription factor unigenes were subjected to three pairwise comparisons which identified 54, 48 and 50 TFs differentially expressed in the F-vs-S, L-vs-F and L-vs-S comparisons (Fig. 5). The bHLH transcription factors were the largest group for F-vs-S and L-vs-F comparison and ERFs were the largest group for the L-vs-S comparison (Fig. 5). These results provide a rich resource for future analysis of the role of transcription factors in lily organogenesis.

### Construction of regulatory network of monoterpene biosynthesis

**Fig. 6:**
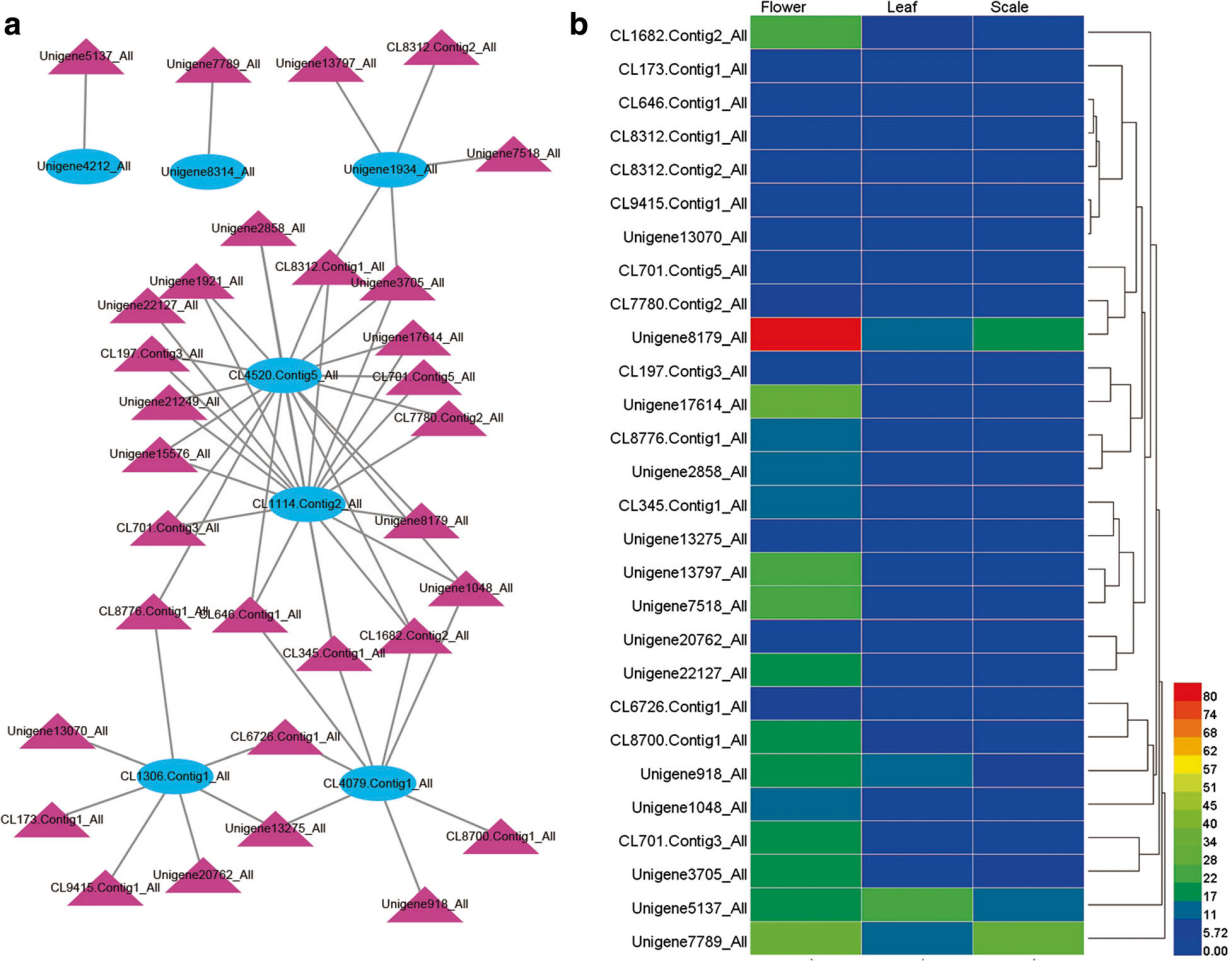
A putative regulatory network from expression profiles of monoterpene biosynthesis genes and transcription factors (TFs). **a** Subnetwork of putative TFs and structural genes related to flower scent volatile biosynthesis. **b** hierarchical clustering of expression profiles of 28 TFs related to flower scent volatile biosynthesis. Gene names are listed together with putative functions in Additional file 11: Table S11

To provide a system view of the regulatory network responsible for controlling monoterpene analysis, networks were extracted based on correlation analysis between 839 putative TFs and seven putative DEGs related to flower fragrance. As a result, 31 putative TFs were identified as potentially involved in regulating the seven putative genes related to production of flower volatiles (Fig. 6a and Additional file 11: Table S11) (*P* < 0.01). These TFs were classified into 13 putative TF families, with the three largest TF families being the bHLH (5 Unigenes), bZIP (5 unigenes) and MYB related (4 unigenes) families (Additional file 11: Table S11). The expression profiles of these TFs potentially related to flower volatile biosynthesis were hierarchically clustered and plotted in a heatmap (Fig. 6b). Three of these (Unigene15576_All, Unigene21249_All, Unigene1921_All) with extremely high expression levels in flowers (RPKM values of 1574.3, 1514.6 and 133.1, respectively) were excluded from the heatmap, but relevant data are shown in Additional file 11: Table S11. Most of those TFs were more highly expressed in flower than in leaf and scale. The expression patterns of all 31 TFs were positively correlated with those of flower scent genes (Additional file 11: Table S11).

### qRT-PCR verification of DEGs identified by RNAseq

**Fig. 7:**
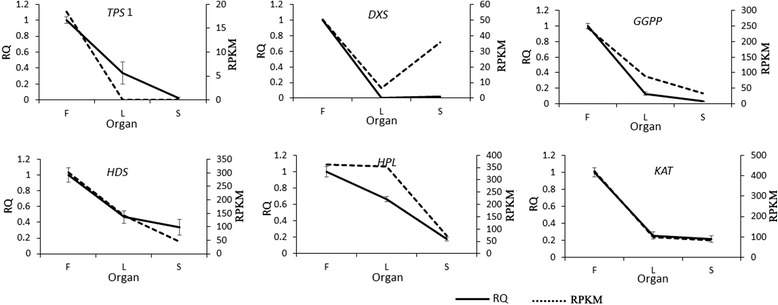
qRT-PCR validations of expression patterns of DEGs involved in flower fragrance. X-axis indicates organs, and y-axis indicates gene expression levels by qPCR (left) and RPKM (right). Solid lines in all plots indicate the relative expression value obtained by qPCR; the dotted lines indicate the RPKM values obtained by RNA-seq

The expression profile of six flower fragrance-related DEGs from lily identified by the RNA-seq approach were tested, using quantitative RT-PCR, including key genes involved in synthesis of monoterpenoids biosynthesis: *TPS, DXS, GGPP, HDS*, and fatty acid derivatives and phenylpropanoid/benzenoid biosynthesis:*HPL* and *KAT*. The comparisons of expression measured by RNA-seq and qRT-PCR in the three organs were largely in agreement (Fig. 7). The expressions of these genes was highest in flowers, although some, such as *HPL* also had high expression in leaves.

### Cloning of TPS genes from cDNA

Terpene synthases (TPS) are that key enzymes that catalyze the last step of the MEP pathway to produce terpenes. There are numerous terpenes and TPSs in plants and small changes in amino acid sequence generate the unique properties and diversity of these important compounds. Primers were designed based on the sequence of transcript CL4520.Contig5_All (accession number: SUB2623518), which had sequence homology with *TPS* genes, and attempts were made to clone the complete cDNA sequence from petal. However, these attempts failed with three different pairs of primers. When the sequence was checked against the NCBI database it was found to be a mixed clone, part of which was highly homologous (91.17% at the cDNA level) to the complete coding sequences of *LhTPS* from ‘Siberia’ (accession number: KF734591), together with a second unrelated open reading frame. Based on the *Lilium* oriental ‘Sorbonne’ sequence information, new primers were designed and a putative *TPS* sequence (NCBI accession: MF401556, designated *LsTPS*) was acquired with an open reading frame (ORF) of 1761 nucleotides, which has 99.55% sequence homology with the TPS coding sequence of transcript CL4520.Contig5_All. Phylogenetic analysis (Additional file 12: Figure S1) based on deduced amino acid sequences showed that *LsTPS* were highly homologous with CL4520.Contig5_All, and with the other two amino acid sequences from *Lilium* ‘Belladonna’ and ‘Siberia’. It was clustered with *LcTPS1*, which is a member of Tps-b group and was able to convert Geranyl diphosphate into *trans-*ocimene [22].

## Discussion

### Profiling of differentially expressed genes in lily organs

The aim of this study was to identify differences in the identities and expression patterns of specific genes and pathways operating in leaves, flowers and bulb scales. To our knowledge, this is the first report of the differentially expressed genes in these three major organs in lily. A large number of new genes and transcription factors involved into photosynthesis, bulb development, flower color and flower scents have been identified. As expected, RNA-seq generated a great deal of information, which will help identify targets to understand the factors controlling organ development and differentiation.

A greater number of DEGs were found in the L-vs-F and L-vs-S comparisons (2685, 2296) than in the F-vs-S group (1709), which indicated that compared to leaf, flower and scale had fewer differentially expressed genes (Fig. 1a). Each organ was also, as expected, characterized by expression of specific classes of genes. In leaves, for example these included genes involved in photosynthesis, carbon metabolism, nitrogen and protein metabolism. In contrast, 21 of the DEGs identified in scales were involved in starch and sucrose metabolism (Additional file 7: Table S7) and the importance of these pathways has previously been demonstrated at the transcriptional level in bulbous plants, such as *Lilium davidii* var. *unicolor* [11], *Gladiolus hybridus* [23] and *Tulipa gesneriana* [24].

### Candidate genes related to flower color, fragrance, photosynthesis and bulb development

In this research, materials were harvested from plants at a stage when leaves were mature and flower color and scents were developing. We believe this is the best time to maximize the gene expression differences between the different organs and mine differentially expressed genes in organs of flower, leaf and scales. Flavonoids are accumulated in pink flowers of lily [25], and genes involved in the flavonoid biosynthesis pathway received special attention in this study with *Lilium* ‘Sorbonne’, which has pink flowers. A total of nine flower color-related genes, *DFR, CHS, CHI, FLS, F3H, LAR, HCT, LDOX, TT7,* were identified. Many of these belong to multi-gene families and several homologs were identified for each gene. Interestingly, a very recent de novo transcriptome sequencing of flower buds at different development phases of *Lilium* ‘Sorbonne’ also identified most of those genes [15], indicating the power of digital gene profiling in gene mining. All of the identified flower color genes have been cloned from different plants such as Arabidopsis and Petunia [26, 27], and an increasing number have been cloned from lily, such as *LhDFRs, LhCHS* [28, 29] and *LhF3S* [30]. *DFR* was identified from the pink petal of ‘Sorbonne’ in this study and *LhDFRs* have also been shown to be expressed in the Asiatic lily ‘Montreux’, which has pink flowers, but not in the Asiatic ‘Connecticut King’, which has yellow flowers [28].

Because oriental lily can emit complicated volatile compounds, it has been regarded as a potential model system for the elucidation of gene products responsible for the synthesis of volatiles [20]. The differential expression of monoterpene synthase genes has been suggested to be a key reason for differences in floral scent [31] and we identified a monoterpene synthase gene (*TPS*) predicted to code for enzymes synthesizing linalool and *trans-*ocimene. Three genes, *LcTPS1, LcTPS2* and *LcTPS3,* have been cloned from *Litsea cubeba* [22], encoding enzymes synthesizing *trans-*ocimene, α-thujene and (+)-sabinene respectively, and other TPS genes, encoding *myrcene synthase* (*LiMys*) and *linalool synthase* (*LiLis*), have also been cloned from *Lilium* ‘Siberia’, ‘Novano’ and *L. regale* [31–33]. Here, we cloned *LsTPS* from *Lilium* ‘Sorbonne’ and predicted the cloned sequence of *LsTPS* might encode enzymes synthesizing *trans-*ocimene by phylogenetic analysis (Additional file 8: Figure S1). Some other genes encoding enzymes involved in production of the terpenoid backbone in the MEP (2- C -methyl- D -erythritol 4-phosphate) pathway, *DXS, GGPP* and *HDS,* were also identified. DXS is a pivotal gene in the first step of MEP pathway and has been extensively studied in tomato, Arabidopsis and grape [34–36], and had been identified and cloned in lily by Johnson et al. [20]. However, functional analysis of these genes is still needed to confirm their role in lily flower scent production and a comparison of expression in scented lilies and those without fragrance [31] should also prove informative.

Unsurprisingly, DEGs participating in photosynthesis were identified as being up-regulated in leaves. Previously, homologous sequences of *psbA* and *atp* has been cloned in a study analyzing the complete plastid genomes of *L. nobilissimum* and *L. longiflorum* [37] and psbA-homologous proteins has been isolated from *L. superbum* [38], and this study has greatly increased the number of gene sequences identified in *Lilium*.

There have been several studies on the variation of carbohydrate compounds during bulblet development [39, 40]. Li et al. [11] revealed the variation in starch and sucrose content at different stages of bulblet initiation and enlargement in *Lilium davidii* var. *unicolor*. However, little is known about changes at the molecular level, especially between underground and above-ground stages. Kawagishi and Miura [41] divided the period of a lily development into four stages, and in this study we used the third stage, corresponding to flower bud expansion to flowering, when both above-ground and underground organs show a vigorous increase in dry weight, and bulbs can be both source and sink [42], underlying the complexities of metabolic activity in bulb scales. We found that putative sucrose- and starch-hydrolysis genes, such as *SS, INV* and *BAM* were down-regulated in scales compared to flowers as reference, and sucrose and starch synthesis genes, such as *SPS* and *SSS* were down-regulated also in scale with leaf as reference, which is consistent with the lack of rapid swelling and bulb growth for plants with 5.5 cm buds used in this study.

### Identification of transcription factors and regulatory network

The largest families of transacting transcription factors modulate plant development [11, 43]. In this study, 839 unigenes were identified as TFs, and 31 of them were identified as potentially involved in regulating production of flower volatiles. Although terpenoid metabolism is very important in biochemical differentiation and tissue function in plants, not many TFs are known to be involved in the regulation of the pathway. In *Solanum lycopersicum,* bHLH and WRKY were identified activating terpene synthase promoters, with MYC synergistically transactivating the *SlTPS5* promoter [44]. and activated distinct terpeniod biosynthesis in *Catharanthus roseus* and *Medicago truncatula* [45]. Ethylene-related TFs have also been implicated in regulating TPSs, for example in *Artemisia annua*, AaERF1 and AaERF2 positively regulate artemisinin production [46] and in Newhall sweet orange CitAP2.10 activates the *CsTPS1* that produces valencene [47]. Based on this comparative information, bHLH, WRKY, ERF/AP2 family members would seem likely candidates for regulating lily flower TPS and studies on other pathways indicate that a complex network may be involved.

In this study, bHLH was the largest TF families in F-vs-S and L-vs-F comparison and in the putative flower volatile biosynthesis network (Fig. 5, Additional file 11: Table S11). CL4520.Contig5_All, a putative *TPS* gene (Additional file 12: Figure S1), were regulated by 17 TFs (Fig. 6a). Four of these were bHLH (Additional file 11: Table S11). However, flower volatile regulation is clearly complex (Fig. 6a), and many TFs, including bHLHs, probably regulate multiple structural genes synchronously during flower development, in concert with other TFs in the network. Candidate TFs have been identified in this study and their role can now be tested. Furthermore, the complete TF database identified from this study has additional potential for improving our understanding of the differential regulation of gene expression during development of leaves, bulbs and flowers.

### Verification of the database

Although high-through sequencing technology has become a powerful tool to identify candidate genes and investigate gene expression patterns [48], further validation is needed, especially for non-model organisms without reference sequences, and for plants with huge genomes. Although in most cases, there was a good correlation between transcript abundance assayed by qRT-PCR and the transcription profile revealed by DGE profiling [11, 16, 49], there have also been reports of inconsistencies between the two methods [50, 51]. In this study, a general agreement was obtained by the two different methods. All verified genes except *HPL* had higher expression level in flower than in leaf and scale. A very recent study revealed *HPL* involvement in protecting the photosynthetic apparatus [52]. We deduce that this gene may play an important role in lily leaf and further functional studies are required to understand its role in lily leaf and flower. A putative error in the assembly of CL4520.Contig5_All was identified during subsequent attempts to clone the cDNA sequence although this did not effect the open reading frame of *LsTPS* and the complete sequence was subsequently obtained. To our knowledge, this is the first report of *TPS* gene from *Lilium* ‘Sorbonne’ and it is proposed that this is important for our understanding of scent production in this species. Overall, the extensive RNA-seq data provides a platform for candidate gene identification in lily organs and elucidation of their function.

## Conclusions

In the present study, DEGs related to lily flower color, flower fragrance, photosynthesis and bulb development were identified by RNA-seq technology. Approximately 11 Mb data were generated for each lily organ, a few thousand DEGs were identified in the comparisons, and hundreds of genes specific for each of the three organs identified. By functional enrichment analysis, genes for floral scent (*TPS, DXS, KAT, HPL* et al.), floral color (*DFR, CHS, CHI* and *FLS* et al.), starch and sucrose metabolism (*SPS, SS, INV, SSS, SBE, AGP* and *BAM*) and photosynthesis (*Psa*, *Psb, Pet and ATP*) were identified. The expression of six floral fragrance-related DGEs showed a similar expression pattern between qRT-PCR results and RPKM values, confirming the value of the data obtained by RNAseq. *LsTPS* was cloned based on the sequence of transcript CL4520.Contig5_All with an ORF of 1761 nucleotides. The lily DGEs identified in this research include transcription factors, flower-specific genes with putative functions in flower color and scent biosynthesis, photosynthesis-related genes in leaf, and starch and sucrose metabolism-related genes in scales, and provide a valuable and informative database for understanding lily organogenesis, mining of important genes, and research into gene function. Based on these results a putative regulatory network for monoterpene biosynthesis is proposed.

## Methods

### Plant materials and RNA extraction

*Lilium* oriental hybrid ‘Sorbonne’ bulbs were planted in the field of the Institute of Landscape Architecture, Zhejiang University (ZJU), China in October, 2013 and they sprouted in March 30th, 2014. Three whole plants were harvested on a sunny day of June 13th at 9 am, 2014 (temperature 24 °C; 41,889.3 Lux) when leaves were mature and flower color and scent were developing and brought to the lab immediately. 5.5 cm flower buds (named F), leaves from the centre of the stem (named L) and inner bulb scales (named S) were collected from each of the three plants. Three biological replicates were used for each organ. Samples were placed in 50 ml tubes and stored at −80 °C until use. The RNA isolation method was as described in Du et al. [53]. A total of nine RNA samples were isolated using a modified CTAB method [54] (three replicates for three organs) respectively. The quality and concentration of RNA were checked using an Angilent 2100. RNA with (RIN) ≥ 7, 28S:18S > 1, OD260/280 ≥ 2, and OD260/230 ≥ 2 were used for sequencing.

### Construction of DGE database

Nine cDNA libraries were constructed respectively based on 9 samples from three replicates of three organs. Details for the construction was as described in Feng et al. [55]. RNA library processing and sequencing via Illumina HiSeqTM 2000 were carried out by staff of the Beijing Genome Institute (BGI) (Shenzhen, China). Clean reads were obtained by data filtering to remove reads with adaptor sequences, more than 10% unknown bases, and those with low quality bases above 50%. The clean reads were mapped to reference sequences of L.-Unigene-All (accession number: SUB2623518), a hybrid assembly comprehensive transcriptome acquired by our lab, using SOAP aligner/SOAP2 [56], and short sequences less than 200 were discarded. The parameters for SOAP aligner/SOAP2 were as follows: option = −m 0 -× 500 -s 40 -l 35 -v 5 -r 2. No more than two mismatches were allowed in the alignments. Once reads passed sequencing saturation and randomness assessment, a digital gene profiling database for each sample was set up.

### Quantification of gene expression

The gene expression level was calculated by using RPKM (Reads per kb per million reads) which eliminates the influence of different sequence lengths and discrepancies due to sequencing depth. The differences in gene expression between samples can be compared directly by comparing RPKM values.

### Screening of differentially expressed genes between two groups

The NOlseq method [57] was used to screen for differentially expressed genes between two groups. Two main steps were conducted: first, the noise distribution was calculated and then DEGs were divided into groups. The expression values for each gene in each group were used to calculate log2 (fold-change) M and the absolute difference value D of all pair conditions (gene expression value was scored as 0.001 for genes not expressed in a sample). For each gene, an average expression value across replicates was used to calculate M and D. Then, all these M/D values were pooled together to estimate the noise distribution. In the case where gene i is differentially expressed between two groups, Gi was set as one, otherwise as 0. This gave the *P* value, the probability of gene i being differentially expressed. Formulae for M, D, P calculation are as follows:

$$ {\mathrm{M}}^{\mathrm{i}}={\log}_2\left({\mathrm{x}}_1^{\mathrm{i}}/{\mathrm{x}}_2^{\mathrm{i}}\right),{\mathrm{D}}^{\mathrm{i}}=\left|{\mathrm{x}}_1^{\mathrm{i}}-{\mathrm{x}}_2^{\mathrm{i}}\right|, $$

$$ \mathrm{P}\left({\mathrm{G}}^{\mathrm{i}}=1|{\mathrm{x}}_1^{\mathrm{i}},{\mathrm{x}}_2^{\mathrm{i}}\right)=\mathrm{P}\left({\mathrm{G}}^{\mathrm{i}}=1|{\mathrm{M}}^{\mathrm{i}}={\mathrm{m}}^{\mathrm{i}},{\mathrm{D}}^{\mathrm{i}}={\mathrm{d}}^{\mathrm{i}}\right)=\mathrm{P}\left(\left|{\mathrm{M}}^{\ast}\right|<\left|{\mathrm{m}}^{\mathrm{i}}\right|,{\mathrm{D}}^{\ast }<{\mathrm{d}}^{\mathrm{i}}\right) $$

In this paper, genes with P greater than 0.8 and M greater than 2 were considered to be differentially expressed between groups.

### Functional analysis of differentially expressed genes

All Unigene sequences were aligned to the protein databases NCBI non-redundant (NR), the Swiss-Prot protein database (Swiss-Prot, in UniProt), the Kyoto Encyclopedia of Genes and Genomes (KEGG, www.genome.jp/kegg/) and the Clusters of Orthologous Groups of proteins (COG, http://www.ncbi.nlm.nih.gov/COG/) (*E* value <10^−5^) by BLASTx. To identify the main biological functions associated with the DEGs, all DEGs were mapped to GO terms in the database, the total numbers of genes for each term were calculated and a hypergeometric test were used. Taking corrected *p*-value ≤ 0.05 as a threshold, significantly enriched GO terms for DEGs were acquired. Then, a Blast2GO program [58] and WEGO software [59] were used to obtain GO functional classifications for DEGs following default parameters. Similarly, all DEGs were mapped to the KEGG database for KEGG pathway enrichment analysis of DEGs.

### Validation DEGs by qRT-PCR

Real-time quantitative RT-PCR was used to validate the expression of a selected set of DEGs. Six primer pairs were designed with the Primer 3.0 (http://primer3.ut.ee/) program, to produce a 150 bp amplicon, based on lily transcriptome database L.-Unigene-All (Additional file 13: Table S12). After a preliminary experiment for evaluation of candidate reference genes, actin (forward primer sequence CACACTGGTGTCATGGTTGG; reverse primer sequence CACAATACC GTGCTCAATTGG), was used as an internal control. Real-time PCR reactions were performed using a 7500 Real Time PCR System (Thermo Fisher Scientific), in a total of 20 μl, with 1 μl cDNA, 0.8 μl forward primer (10 μM), 0.8 μl reverse primer (μM), 0.4 μl ROX, and 10 μl SYBR® Premix Ex TaqTM II(2×). The cycling conditions were as follows: 95 °C for 3 min, 40 cycles of 95 °C for 30 s, 55 °C for 30 s, and 72 °C for 1 min. Melting curves for each PCR product were analyzed at 95 °C for 15 s, cooling to 54 °C for 1 min, and then gradually heated at 0.1 °C/s to a final temperature of 95 °C. Relative quantitation (RQ) was calculated using the 2 − ΔΔCt method. Three RNA isolations and triplicate RT-PCR runs were implemented for each sample for biological and technical replication.

### Cloning and nucleotide analysis of *TPS* gene

Gene cloning primers were designed taking sequences of transcript CL4520.Contig5_All from L.-Unigene-All as template. Forward primer (ATGGCAGCTATGAGCTGT) and reverse primer (TCATTCCAATGGGACATTATTG) were synthesized by Sangon Biotech. The PCR was performed with a Gene Amp kit (Transgen Biotech, China) according to the manufacturer’s instructions, in a total of 50 μl, with 4 μl of petal cDNA, 1 μl forward primer (10 μM), 1 μl reverse primer (μM), 5 μl TransTaq-T buffer, 4 μl 2.5 mM dNTPs, and 1 μl TransTaq-T DNA Polymerase. The cycling conditions were as follows: 94 °C for 5 min, 35 cycles of 94 °C for 30 s, 55 °C for 30 s, and 72 °C for 1 min. Following detection and excision from the agarose, the fragment was subsequently cloned into pMD 19-T vector and transformed into Trans5α *E. coli* cell according to the manufacturer’s protocol (Transgen Biotech, China). Three clones screened and vetted for amplicons of appropriate size were sequenced by Sangon Biotech. Multiple sequence alignment of plant *TPS* gene was performed using DNAMAN 8.0 (Lynnon Biosoft, USA) and a phylogenetic tree was constructed using MEGA5 with a bootstrap replication of 1000.

### Identification of transcription factor

Transcriptome database L.-Unigene-All was used for transcription factor identification against the Plant Transcription Factor Database PlnTFDB (http://plntfdb.bio.uni-potsdam.de/v3.0/downloads.php) using BLASTX with an E-value cut-off of ≤ 10^−5^.

### Construction of gene expression profiles and gene co-expression network

HemI 1.0.3.7 [60] was used to construct a heatmap of DGEs related to monoterpene biosynthesis and expression profiles of transcription factors. Pearson correlation coefficient (PCC) were calculated between the two genes from two data sets, DEGs related to flower fragrance (Additional file 8: Table S8) and the expressed identified TFs. TFs with |PCC| ≥ 0.95 (*p*<0.01) were selected for co-expression network construction from the DEGs related to flower fragrance. The networks were visualized using Cytoscape _v.3.3.0 [61].

## Additional files

Additional file 1: Table S1. Mapping reads alignment results for the discarded leaf sample. (XLSX 10 kb)

Additional file 2: Table S2. Original data for RPKM values and functional annotation of all transcripts. (XLS 33444 kb)

Additional file 3: Table S3. Review of common unigenes in flower, leaf and scale. (XLSX 13 kb)

Additional file 4: Table S4. Flower-specific genes. (XLSX 38 kb)

Additional file 5: Table S5. Leaf-specific genes. (XLSX 47 kb)

Additional file 6: Table S6. Scale-specific genes. (XLSX 36 kb)

Additional file 7: Table S7. Differentially expressed genes involved in flavonoid biosynthesis. (XLSX 17 kb)

Additional file 8: Table S8. Differentially expressed genes related to flower fragrance. (XLSX 16 kb)

Additional file 9: Table S9. Differentially expressed genes involved in photosynthesis. (XLSX 21 kb)

Additional file 10: Table S10. Differentially expressed genes involved in metabolism of starch and sucrose. (XLSX 18 kb)

Additional file 11: Table S11. Review of structural genes related to flower fragrance and their highly correlated transcript factors. (XLSX 23 kb)

Additional file 12: Figure S1. Alignment of deduced amino acid sequences of LsTPS with sequences from different fragrant plants. Constructed using MEGA5 with a bootstrap replications of 1000. (PDF 61 kb)

Additional file 13: Table S12. Primer sequences used for qRT-PCR validation of RNA-seq data. (PDF 135 kb)

## Abbreviations

AGP: ADP-glucose pyrophosphorylase
BAM: β-amylase
CHI: Chalcone isomerase
CHS: Chalcone synthase
DEGs: Differentially expressed genes
DFR: Dihydroflavonol 4-reductase
DGE: Digital gene expression
DXS: 1-deoxy-D-xylulose-5-phosphate synthase
F3H: Flavanone 3-hdroxylase
FLS: Flavonol synthase
F-vs-S: Flowers-vs-scales
GGPP: Geranylgeranyl pyrophosphate
GO: Gene Ontology
HCT: Shikimate O-hydroxycinnamoyltransferase
HDS: 4-hydroxy-3-methylbut-2-en- 1-yl diphosphate
HPL: Hydroperoxide lyase
INV: Invertase
KAT: 3-ketoacyl-CoA thiolase
KEGG: Kyoto Encyclopedia of Genes and Genomes
LAR: Leucoanthocyanidin reductase
LDOX: Leucoanthocyanidin dioxygenase
L-vs-F: Leaf-vs-flower
L-vs-S: Leaves-vs-scales
qRT-PCR: quantitative real time PCR
RPKM: Reads per kb per million reads
SBE: starch branching enzyme
SPS: Sucrose phosphate synthase
SS: Sucrose synthase
SSS: Starch synthase
TPS: terpene synthase
TT7: Flavonoid 3′-monooxygenase
