# Supplementary figures and images for: Identification of differentially expressed genes in flower, leaf and bulb scale of Lilium oriental hybrid ‘Sorbonne’ and putative control network for scent genes
Source: BMC Genomics. 2017 Nov 22;18:899. doi: 10.1186/s12864-017-4303-4 (PMC5700745; doi:10.1186/s12864-017-4303-4)

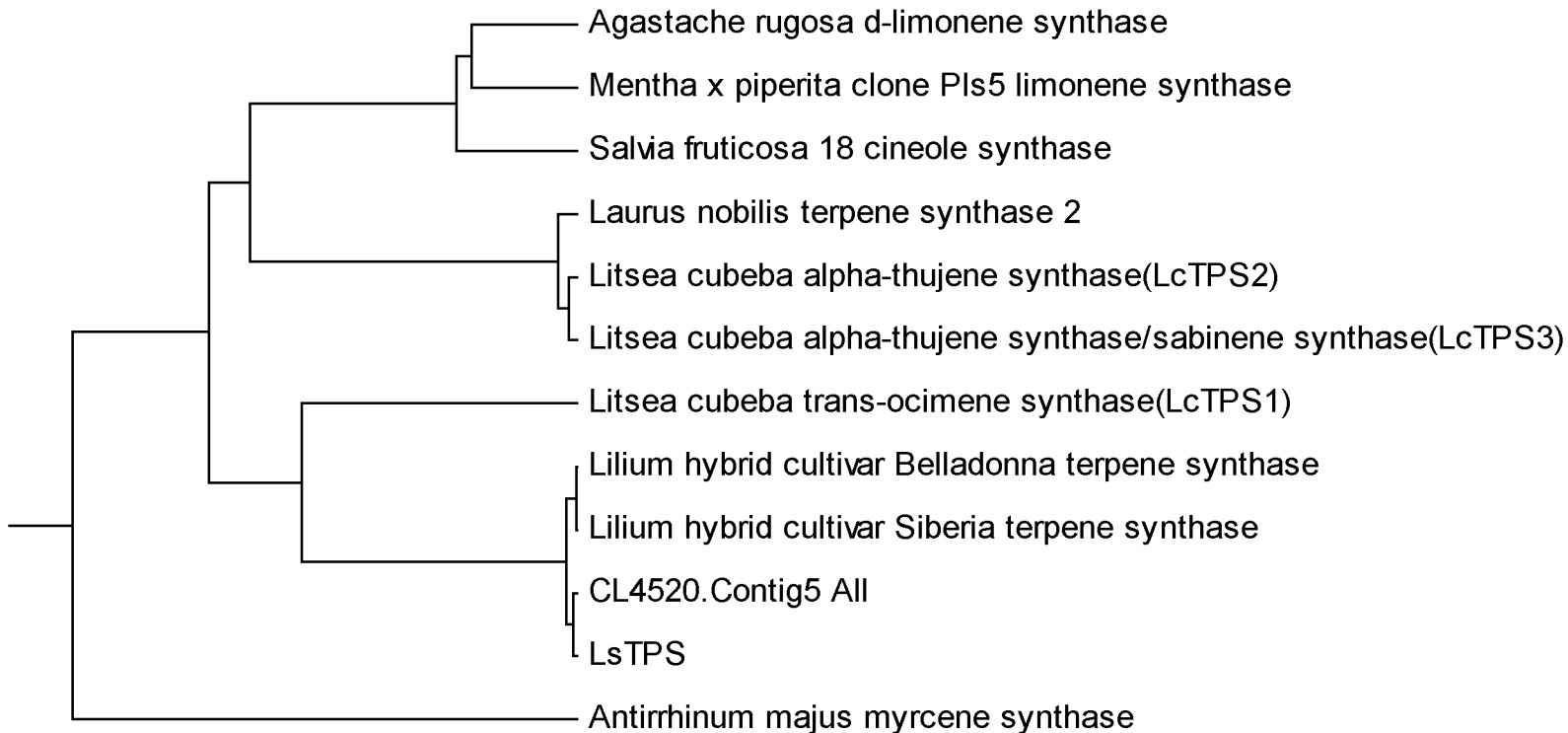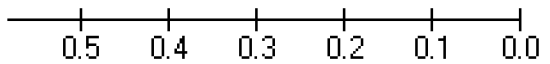

Supplement: Supplementary file 12 — Alignment of deduced amino acid sequences of LsTPS with sequences from different fragrant plants. Constructed using MEGA5 with a bootstrap replications of 1000. (PDF 61 kb) [file 12864_2017_4303_MOESM12_ESM.pdf]
