# Supplementary material for: Identification of differentially expressed genes in flower, leaf and bulb scale of Lilium oriental hybrid ‘Sorbonne’ and putative control network for scent genes
Source: BMC Genomics. 2017 Nov 22;18:899. doi: 10.1186/s12864-017-4303-4 (PMC5700745; doi:10.1186/s12864-017-4303-4)
Supplement: Supplementary file 13 — Primer sequences used for qRT-PCR validation of RNA-seq data. (PDF 135 kb) [file 12864_2017_4303_MOESM13_ESM.pdf]

Additional file 13: Table S12. Primer sequences used for qRT-PCR validation of RNA-seq data

Transcript ID	Gene	Putative protein	GI No.	Primer pairs sequence (5’-3’)
CL1114.Contig2_All	*KAT*	Benzyl-CoA	212723032	F:TGCAGTGAAATCTGCTGGTC<br>R: GCAAAAGCCTCGTTGATCTC
CL1306.Contig1_All	*GGPPS*	Geranylgeranyl pyrophosphate synthase	357122245	F:AAGGATCTCGCCACAGACAA<br>R:GCCTTCTCCACGTTGAATCC
CL4520.Contig5_All	*TPS*	Trans-ocimene synthase	339744294	F:GAGACTCGCAAGGGTTCAAG<br>R:ATCCTACGGTGCAATGGAA
Unigene4212_All	*HPL*	Hydroperoxide lyase	115443737	F:AGGTTTACAGGTGCGAAAGG<br>R:GAGAATCTGAGCCACAAGCA
Unigene8314_All	*DXS*	1-deoxyxylulose-5-phosphate synthase	255583239	F:GCTGCGACTGTCCTCTCTCT<br>R:TTGACACGAACATTGGATCA
CL4079.Contig1_All	*HDS*	4-hydroxy-3-methylbut-2-en-1-yl diphosphate synthase	470117782	F:GTCTTAATAAGGGCGTGGCG<br>R:CCACCTCGTCACTCTCCTTT
